# Annual Profile of Patients at a Child and Adolescent Mental Health Day Hospital in Chile

**DOI:** 10.1192/j.eurpsy.2026.11171

**Published:** 2026-07-31

**Authors:** L. Blasco, T. Ferrer, I. Campusano-Castañeda

**Affiliations:** 1 Parc Sanitari Sant Joan de Déu, Sant Boi de Llobregat, Spain; 2 Salud Mental y Psiquiatría, Hospital Dr. Exequiel González Cortés, Santiago de Chile, Chile

## Abstract

**Introduction:**

Mental Health Day Hospitals (MHDH) are intermediate facilities that provide intensive daily care for children and adolescents, allowing them to return home at the end of the day. Their multidisciplinary approach seeks clinical stabilization, improved functioning, and the prevention of hospitalizations. In Chile, approximately twelve MHDH units serve the child and adolescent population. The Dr. Exequiel González Cortés Hospital operates one of these units, focusing on adolescents with severe psychiatric disorders under a community- and rights-based model.

**Objectives:**

The study describes the clinical and sociodemographic characteristics of patients treated at the Dr. Exequiel González Cortés Mental Health Day Hospital over a one-year period (July 2024–June 2025).

**Methods:**

A retrospective review of admission records was conducted. Patient data were analyzed using descriptive statistics and prevalence estimates in Excel.

**Results:**

A total of 49 adolescents were admitted: 20.4% male and 79.6% female, with 20.5% of the latter in transition to male gender. Regarding the primary diagnosis, 26.5% presented with affective disorders: 6.1% with treatment-resistant depression, 6.1% with severe depression with psychosis, 6.1% with bipolar disorder, and 8.2% with depression associated with high suicide risk. None of the patients were diagnosed with substance use disorders. The most prevalent diagnosis was post-traumatic stress disorder (30.6%), followed by eating disorders (16.3%). Personality disorders and autism spectrum disorder were each present in 8.2% of cases. Schizophrenia was diagnosed in 0.2% of patients, and 0.6% presented with other disorders.

**Conclusions:**

The findings highlight a high prevalence of post-traumatic stress disorder, affective disorders, and eating disorders, reflecting the diversity and complexity of the cases treated. These results provide valuable insights to optimize and adapt interventions according to patient needs, thereby strengthening the therapeutic model of Mental Health Day Hospitals in Chile.

**Disclosure of Interest:**

None Declared

